# Shortcuts in biodiversity research: What determines the performance of higher taxa as surrogates for species?

**DOI:** 10.1002/ece3.2736

**Published:** 2017-03-18

**Authors:** Neil Rosser

**Affiliations:** ^1^ Department of BiologyUniversity of YorkYorkUK

**Keywords:** ants, beetles, community composition, higher taxa, niche conservation, species richness, surrogates

## Abstract

Biodiversity research is often impeded by the time and resources required to identify species. One possible solution is to use higher taxa to predict species richness and community composition. However, previous studies have shown that the performance of higher taxa as surrogates for species is highly variable, making it difficult to predict whether the method will be reliable for a particular objective. Using 8 independent datasets, I tested whether higher taxa accurately characterize the responses of beetle and ant communities to environmental drivers. For each dataset, ordinations were carried out using species and higher taxa, and the two compared using the Procrustes *m²* statistic (a scale‐independent variant of Procrustes sum of squares). I then modelled the relationship between five hypothesised explanatory variables and 1) Procrustes *m²*, and 2) the coefficient of determination (*R²*) for the correlation between richness of species and higher taxa. The species to higher taxon ratio, community structure, beta diversity, completeness of sampling, and taxon (beetles or ants) were all significant predictors of *m²*, together explaining 88% of the variance. The only significant predictor of *R²* was the species to higher taxon ratio, which explained 45% of the variance. When using higher taxa to predict community composition, better performance is expected when the ratio of species to higher taxa is low, in communities with high evenness and high species turnover, and when there is niche conservation within higher taxa. When using higher taxa to predict species richness, effective surrogacy can be expected when the species to higher taxon ratio is very low. When it is not, surrogacy performance may be strongly influenced by stochastic factors, making predictions of performance difficult.

## 1 Introduction

The time and resources required to identify species impair the assessment and monitoring of biodiversity, especially in very diverse and/or poorly known taxa such as insects, plants, and fungi. Consequently, a large body of research has been devoted to identifying surrogates for species‐level data, such as morphospecies, indicator groups, and environmental variables (Beccaloni & Gaston, 1995; Beier & de Albuquerque, 2015; Oliver & Beattie, 1996; Rodrigues & Brooks, 2007). A further possibility is to use higher taxa in place of species, for example genera, families, or even orders. This approach has received considerable attention in the conservation literature because it is much easier to identify and count the number of higher taxa than it is the number of species, thereby facilitating the prioritization of sites on the basis of species richness (Balmford, Green, & Murray, 1996; Balmford, Jayasuriya, & Green, 1996; Gaston & Williams, 1993). Because higher taxa retain some information on the identity of organisms being studied (Gaston & Williams, 1993), they can also be used to characterize communities and their responses to natural and anthropogenic environmental drivers. This is often known as “taxonomic sufficiency” and has been studied extensively within the context of biomonitoring freshwater and marine ecosystems (Bates, Scott, Tobin, & Thompson, 2007; Ellis, 1985; Heino & Soininen, 2007; Melo Carneiro, Bini, & Rodrigues, 2010; Warwick, 1988; Wright, Chessman, Fairweather, & Benson, 1995). However, many of these studies have produced conflicting results, making generalizations about the implications of taxonomic sufficiency difficult (Jones, 2008).

There have been far fewer tests of taxonomic sufficiency in terrestrial ecosystems, but those that exist have produced similarly discordant findings. While some studies have suggested that species‐level ordinations or beta diversity is adequately predicted by higher taxa (Caruso & Migliorini, 2006; Pik, Dangerfield, Bramble, Angus, & Nipperess, 2002; Pik, Oliver, & Beattie, 1999; Timms, Bowden, Summerville, & Buddle, 2013), others have drawn negative conclusions (Basset et al., 2004; Grimbacher, Catterall, & Kitching, 2008; Nahmani, Lavelle, & Rossi, 2006; Prinzing, Klotz, Stadler, & Brandl, 2003). This conflict is mirrored in studies testing the correlation between richness of species and higher taxa. Some have concluded that families or orders can predict species richness (Gaston & Blackburn, 1995; Prinzing et al., 2003), while others have shown that even genera can fail to predict species richness (Andersen, 1995; Ferla, Taplin, Ockwell, & Lovett, 2002; Prance, 1994; Rosser & Eggleton, 2012).

Recently, attempts have been made to improve our understanding of the factors influencing the ability of higher taxonomic data to predict species‐level patterns (Bevilacqua, Terlizzi, Claudet, Fraschetti, & Boero, 2012). This is important because it would allow researchers to more accurately predict when higher taxa might be suitable for a given objective. The most frequently recognized influence of the surrogacy relationship is the ratio of species to higher taxa (Andersen, 1995). Indeed, the nested structure of the taxonomic hierarchy means that species and higher taxa must always exhibit some degree of correlation (Gaston, 2000). The magnitude of variations in species richness and community composition across a study area has also been hypothesized to affect the surrogacy relationship, because greater variations in species richness or composition will be more likely to be reflected by higher taxa. Therefore, higher taxa may be better surrogates at broader spatial or temporal scales (Balmford, Green, et al., 1996; Rosser & Eggleton, 2012; but see Andersen, 1995). However, to my knowledge this prediction has never been explicitly tested. Recently, Neeson, Van Rijn, and Mandelik (2013) used a mathematical model to show that evenness of community structure can also influence the strength of the correlation between richness of species and higher taxa, with weaker correlations in more even communities. In addition, they found spurious correlations (i.e., mathematical artifacts) can be produced when species have not been well sampled (i.e., before species accumulation curves have reached an asymptote). Finally, whether higher taxa reflect species may depend on the extent to which species niches are conserved within higher taxa; i.e., whether higher taxa form ecologically coherent groups (Warwick, 1993).

Here, I test whether higher taxa can be used in place of species to characterize ant and beetle communities and their responses to environmental drivers. I analyze eight large and independent datasets collected at local scales in six tropical and two temperate countries, thus allowing greater generalizations than many previous studies. I then use the results, along with previously published correlations for the relationship between higher taxa and species richness, to test whether (i) the species to higher taxon ratio, (ii) community structure, (iii) species turnover, (iv) completeness of sampling, and (v) taxon (beetles or ants) determine the strength of the surrogacy relationship.

## 2 Methods

### 2.1 Data

Species abundance data from standard biodiversity surveys of leaf litter dwelling beetles and ants were obtained from databases of the Soil Biodiversity Group at the Natural History Museum, London. The datasets were described in detail elsewhere (Rosser & Eggleton, 2012). Beetle surveys were conducted in Chile, Malaysian Borneo, and the UK. Survey sample units comprised 100‐m‐long transects. These transects were sampled using 1 m² quadrats at 7‐meter intervals, which were then pooled to make up a single sampling unit. The UK dataset comprised 12 samples taken from a single wooded site over the course of 1 year (one sample per month). Data from Chile consisted of 14 samples collected from four different habitat types in Aysén (two deciduous forests, temperate rain forest, and steppe‐edge forest). Data from Borneo comprised two datasets collected in Sabah over 2 years. In the first year, five samples were collected from primary rain forest, small fragments of rain forest, and an oil palm plantation. In the second year, a further five samples were collected along a gradient from primary undisturbed rain forest to entirely deforested land. Generic data were not available for the year two dataset, and so this was only used to test the utility of families as surrogates.

Ant surveys were conducted in Belize, Cameroon, Gabon, Ghana, and peninsula Malaysia. Data from Cameroon and Ghana were collected using the method described above, with sample units comprising 100‐m transects. In Cameroon, seven samples were taken from three undisturbed forests and two deforested areas. In Ghana, 30 samples were taken from primary forest, degraded primary forest, secondary forest, and coconut plantations. Sample units for Belize (79 samples), Gabon (39 samples), and Malaysia (138 samples) comprised randomly distributed 1 m² quadrats taken from primary forest.

### 2.2 Statistical analysis

Statistical analyses were carried out in R (R Core Team 2013) using the packages *vegan* (Oksanen et al., 2013), *nlme* (Pinheiro, Bates, DebRoy, & Sarkar, 2016), *lme4* (Pinheiro et al., 2016), and *MuMIn* (Bartoń, 2016). I used detrended correspondence analysis (DCA) to test whether ordinations carried out using species can be predicted by ordinations carried using higher taxa (genera or families for beetles, and genera or subfamilies for ants). DCA is an extension of correspondence analysis (CA) designed specifically for use with long ecological gradients and to tackle artifacts produced by CA (Hill & Gauch, 1980). I employ DCA here because it has the convenient property that units of DC axes correspond to the average standard deviation of species turnover (sd). Specifically, a 50% change in species composition occurs between sites separated by 1 sd, and sites separated by ≥4 sd share few or no taxa (Hill & Gauch, 1980). Therefore, axis length (i.e., the distance between the two most distant samples) reflects the change in species composition. This allows a direct test of the hypothesis that higher taxa are better surrogates at broader spatial or temporal scales because the magnitude of variations in species richness and community composition is higher (Rosser & Eggleton, 2012). While DCA has been the subject of considerable controversy, strong arguments can be made in favor of its use as oppose to other competing ordination methods (ter Braak & Šmilauer, 2014; Jackson & Somers, 1991), and it continues to be employed in community ecology (Jew, Loos, Dougill, Sallu, & Benton, 2015). I do not anticipate that the criticisms of DCA (e.g., the artificial removal of the “arch effect”) should invalidate the conclusions presented here. Abundance data were log(*x *+* *1) transformed prior to DCA. For each dataset, I conducted ordinations for species, genus, and subfamily/family data. I then tested the correlation between sample unit ordination scores for species and those of higher taxa, using Pearson's product moment correlation and the coefficient of determination (*R²*). I do not report *p* values because the statistical dependence between species and higher taxa means that they are unreliable (Gaston, 2000). I used Procrustes analysis to compare ordinations generated with species and higher taxa (Schönemann and Carroll (1971); Gower, 1971). Procrustes analysis uses rotation and uniform scaling (expansion and contraction) to match the samples in one ordination to the other, such that the squared differences between samples are minimized. I report the Procrustes *m²* statistic, a scale‐independent goodness‐of‐fit statistic based on the sum of the squared deviations, which varies from 0 to 1, with 0 indicating that the results of ordinations are very similar and 1 indicating that they are very different.

I then used the eight datasets to test which variables predict (i) the correlation between species richness and richness of higher taxa and (ii) the similarity between ordinations conducted using species and higher taxa (measured as *m²*). The correlations between species richness and higher taxa for these datasets were previously published in Rosser and Eggleton (2012), and *R²* values for those correlations are shown in Table 1. I applied a linear mixed effect model with either *R²* or *m²* as the response variable and with one categorical and four continuous explanatory variables. The response variables *R²* and *m²* are bounded between zero and one and so were logit‐transformed (Warton & Hui, 2011). The explanatory variables were (i) the species to higher taxon ratio (SHR), (ii) the proportional abundance of the most common species (*D*_*BP*_; the Berger–Parker index of evenness (Berger & Parker, 1970)), (iii) the degree of species turnover along the primary environmental gradient affecting the community (as estimated by the length of the first DCA axis [DC1]), (iv) the proximity to asymptote of the species accumulation curve for the dataset, and (v) the taxon, i.e., beetles or ants. The proximity to asymptote of the species accumulation curve for each dataset was estimated by first dividing the mean species richness at each point in the curve by the mean richness at the highest point in the curve. This scales it between zero and one, making the curves from different datasets directly comparable, even though the sampling units are not always the same size. Distance to asymptote was then given as the difference in scaled species richness between the final two points in the curve (i.e., the slope at the flattest point).

**Table 1:** The columns entitled “correspondence between species and higher taxa” show (i) the correlations between DC axes calculated using species and higher taxa, measured as *R²*, (ii) the similarity of DCA ordinations carried out using species and higher taxa (Procrustes *m²* statistic), and (iii) the correlation between species richness and higher taxa richness (Richness *R²*). Procrustes *m²* and Richness *R²* were subsequently modeled as a function of SHR (the species to higher taxon ratio), *D*_*BP*_ (the proportional abundance of the most abundant species in the dataset, i.e., community evenness), DC1 length (i.e., species turnover), the completeness of species sampling in the dataset (“scaled asymptote”), and taxon (i.e., beetles or ants)

Dataset	Surrogate	Correspondence between species and higher taxa						Predictors of Procrustes *m²* and Richness *R²*					MRPP test	
		DC1 *R²*	DC2 R²	DC3 R²	DC4 R²	Procrustes *m²*	Richness *R²*	SHR	*D*_*BP*_	DC1 axis length	Scaled asymptote	Taxon	*A*	*p*
Borneo (y1)	Genera	.99	.02	‐	‐	0.39	.99	1.93	0.07	10.25	0.15	Beetles	0.11	**.00**
	Families	.97	.13	.02	‐	0.30	.87	8.23					0.02	**.01**
Borneo (y2)	Families	.99	.38	.34	‐	0.17	.91	13.29	0.04	6.54	0.14		0.02	**.07**
Chile	Genera	.98	.01	.06	.20	0.17	.97	1.15	0.16	5.10	0.04	Beetles	0.01	.30
	Families	.90	.03	.00	.04	0.31	.55	4.13					−0.02	.68
UK	Genera	.95	.27	.01	.07	0.36	.97	1.45	0.74	2.00	0.04	Beetles	0.01	**.06**
	Families	.78	.19	.01	.11	0.39	.09	6.27					0.01	**.03**
Belize	Genera	.05	.01	.00	.04	0.74	.87	2.90	0.30	5.36	0.00	Ants	0.02	.13
	Families	.00	.04	.04	.00	0.89	.51	10.50					0.00	.46
Cameroon	Genera	.55	.10	.05	.70	0.61	.61	3.36	0.18	2.03	0.05	Ants	−0.01	.59
	Families	.00	.05	.01	.14	0.64	.31	13.88					0.01	.14
Gabon	Genera	.00	.11	.02	.03	0.77	.64	3.33	0.24	5.46	0.01	Ants	0.03	.14
	Families	.05	.06	.00	.02	0.95	.03	12.50					0.01	.11
Ghana	Genera	.62	.05	.01	.01	0.63	.81	4.07	0.15	3.13	0.01	Ants	0.00	.41
	Families	.10	.00	.00	.00	0.81	.38	20.78					0.01	.12
Malaysia	Genera	.43	.08	.25	.00	0.65	.91	2.85	0.20	4.75	0.00	Ants	0.01	.21
	Families	.03	.01	.00	.02	0.90	.41	13.88					0.01	.12

The final two columns refer to the MRPP test: *A* is an estimate of the proportion of the dissimilarities explained by group identity, and *p* is the significance value of the test. Significant (*p *<* *0.05 and close to significant results (*p *<* *0.1) are highlighted in bold.

*R²* and *m²* are estimated twice for each dataset (for both species/genera and species/higher taxa). To control for this nonindependence, I estimated a random intercept for each dataset (*a*_*j*_) with variance ($\sigma_{a}^{2}$). The resulting model could thus be written as

$$\begin{array}{cc} \log\left(\frac{Y_{ij}}{\left[1-Y_{ij}\right]}\right)=\; & \alpha +\beta_{1}{\text{SHR}}_{ij}+\beta_{2}{\text{DBP}}_{ij}+\beta_{3}{\text{DCA}}_{ij}+\beta_{4}{\text{asymptote}}_{ij}\\ & +\beta_{5}{\text{taxon}}_{ij}+a_{j}+\varepsilon_{ij} \end{array}$$

where

*a_j_*~*N*(0, $\sigma_{a}^{2}$) and $\varepsilon_{ij}$~*N* (0, $\sigma^{2}$)

and where index *i* is the observation and *j* is the dataset. In all but one dataset (Borneo Y2), *R²* and *m²* were estimated for both genera and subfamilies/families, thus for modeling purposes *n* = 17. I standardized the continuous predictors to obtain comparable coefficients independent of measurement unit, by subtracting the mean from each variable's values and dividing the result by the standard deviation. I checked for multicollinearity by examining the correlation coefficient between all combinations of predictor variables. The correlations ranged from moderately positive (*R =* 0.66; DCA1 length and asymptote) to moderately negative: (*R = *−0.53; DCA1 length and *D*_*BP*_.). To validate the model, I used plots of the normalized residuals against fitted values and the explanatory variables. To test the significance of explanatory variables, I fitted the full model using maximum‐likelihood (ML) estimation and then dropped each explanatory variable in turn and compared the reduced model with the full model using likelihood ratio tests. I used marginal *R²* as a measure of the variance explained by the full model (Nakagawa & Schielzeth, 2013).

To test whether species within higher taxa tend to co‐occur, I applied a multiple response permutation procedure (MRPP) to each dataset. MRPP is a nonparametric test that compares the mean pairwise dissimilarities in species composition within sampling units (in this case, higher taxa) with those of random aggregations (of species). If species within higher taxa tend to cooccur (as expected by niche conservatism), the within‐group dissimilarities should be less than the random aggregation dissimilarities.

## 3 Results

### 3.1 Correspondence between ordinations using species and higher taxa

**Figure 1:**
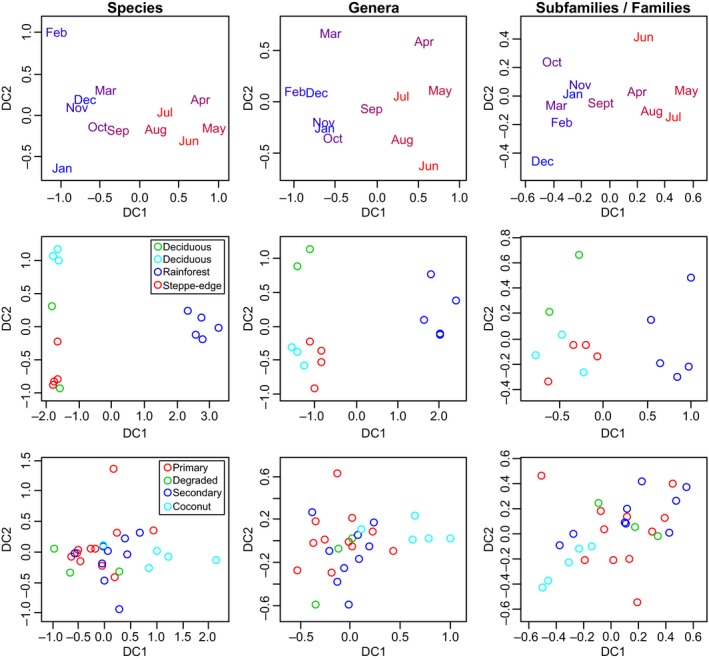
Example ordination plots generated using species and higher taxa. Top panel: beetles from a forest in the UK. One sample per month was taken throughout the year. To aid interpretation, the samples are colored from red to blue according to temporal similarity. Middle panel: beetles sampled from temperate forest habitats in Chile. Bottom panel: ants sampled from forested habitats and a coconut plantation in Ghana

The results of Pearson's product moment correlations between DCA axes from ordinations carried out with species and higher taxa are shown in Table 1. For beetles, DC1 from species‐level ordinations was very strongly correlated with DC1 from ordinations carried out with genera (*R²* = 0.95–0.99) and families (*R²* = 0.78–0.99). For ants, the strength of the correlation between DC1 from species‐level ordinations and DC1 from genera‐level ordinations was variable and always much weaker than for beetles (*R²* = 0–0.62), and the correlation with DC1 from subfamily‐level ordinations was always low (*R²* = 0–0.1). Correlations between other axes from species‐level ordinations were at most weakly correlated with those from ordinations using higher taxa. For beetles, the Procrustes *m²* statistic indicated that ordinations using species and higher taxa were only moderately similar (*m²* = 0.17–0.39), and for ants, ordinations using species and higher taxa were very different (*m²* = 0.61–0.95). Example ordination plots generated by DCA are shown in Figure 1.

### 3.2 Testing predictors of the strength of the surrogacy relationship

I then modeled the variation in (i) the correlation between species richness and richness of higher taxa (“Richness *R²*”) and ii) the correspondence between ordinations conducted using species and higher taxa (Procrustes *m²*), using the explanatory variables SHR, *D*_*BP*_, DC1 length, asymptote, and taxon. The values for both response and explanatory variables are shown in Table 1. Model coefficients and their significance are presented in Table 2, along with marginal *R²* values. The only significant predictor of Richness *R²* was the species to higher taxon ratio (SHR), which was positively related, and the model explained 45% of the variance in Richness *R²*. In contrast, *m²* was significantly predicted by all explanatory variables, with the model explaining 88% of the variance in Procrustes *m²*. SHR, *D*_*BP*_, and DC1 length were all positively related to *m²*. Asymptote (i.e., the completeness of species sampling) was negatively related to *m²*, and beetles had significantly lower *m²* values than ants. For beetles, MRPP (Table 1) found evidence for species within higher taxa tending to occur in three of seven cases (*p* < .05) or five of seven cases (*p* < .1). In contrast, no such associations were observed for ants (*n* = 10), even for *p* < 0.1.

**Table 2:** Coefficients and associated likelihood ratio tests for models predicting (i) the correlation between species richness and richness of higher taxa (Richness *R²*) and (ii) the similarity between ordinations conducted using species and higher taxa (Procrustes *m²*) as functions of SHR (the species to higher taxon ratio), *D*_*BP*_ (i.e., community evenness), DC1 length (i.e., species turnover), the completeness of species sampling in the dataset (“scaled asymptote”), and taxon (i.e., beetles or ants)

Response	Predictor	Coefficient ± standard error	χ²	Significance	Marginal *R²*
Richness *R²*	SHR	−1.2 ± 0.38	7.72	*p *<* *.01	.45
	*D*_*BP*_	−0.55 ± 0.5	1.15	ns	
	DC1 axis length	0.03 ± 0.52	0.00	ns	
	Scaled asymptote	0.51 ± 0.64	0.63	ns	
	Taxon beetles	0.55 ± 1.16	0.22	ns	
Procrustes *m²*	SHR	0.4 ± 0.11	10.43	*p *<* *.01	.88
	*D*_*BP*_	0.55 ± 0.14	10.90	*p *<* *.001	
	DC1 axis length	0.72 ± 0.14	14.62	*p *<* *.001	
	Scaled asymptote	−0.39 ± 0.18	4.19	*p *<* *.05	
	Taxon beetles	−2.14 ± 0.32	18.66	*p *<* *.001	

## 4 Discussion

Several previous studies have examined the potential for higher taxa to act as surrogates for species when studying assemblage composition of terrestrial invertebrates. While some have supported their use (Brennan, Ashby, Majer, Moir, & Koch, 2006; Caruso & Migliorini, 2006), others have drawn negative conclusions (Basset et al., 2004; Nahmani et al., 2006). In Australia, using ants to monitor environmental change is commonplace, and previous work has suggested that assemblages of higher taxa could be used in place of species for these purposes (Nakamura, Catterall, House, Kitching, & Burwell, 2006; Pik et al., 1999, 2002; Schnell, Pik, & Dangerfield, 2003). However, the results presented here for five ant datasets from Africa, South Asia, and Central America indicate very little correspondence between ordinations carried out using higher taxa and species; ordination axes were at best moderately correlated between species and higher taxa, and Procrustes *m²* values were uniformly high.

To my knowledge, two previous studies have used beetles to test whether patterns of species assemblage composition are reflected by higher taxa. Timms et al. (2013) analyzed three datasets from Canadian forests subject to disturbance treatments and found that both beetle genera and families provided suitable surrogates. In contrast, Grimbacher et al. (2008) found that species assemblage responses to deforestation in Australian tropical wet forest were only weakly apparent at family level (genus‐level data was not analysed). For the three tropical and temperate datasets analyzed here, I found that the first DCA axis structuring communities was very strongly correlated between species and genera, and strongly or very strongly correlated between species and families. However, other ordination axes were uncorrelated. Accordingly, Procrustes *m²* values indicated moderate correspondence between ordinations. Therefore, for these datasets, higher taxa would appear to be suitable only for monitoring the response of a community to a clearly dominant environmental variable.

The discrepancies between these results and previously published studies on beetles and ants are similar to those observed in tests of taxonomic sufficiency in aquatic systems (Jones, 2008). Studies testing the correlation between richness of species and higher taxa have also produced conflicting results, variously recommending families, genera, or species as the optimal taxonomic resolution (Balmford, Lyon, & Lang, 2000; Kallimanis et al., 2012; Rosser & Eggleton, 2012). Given these inconsistencies, I sought to discern what factors might influence the strength of the surrogacy relationship. The most frequently discussed predictor of surrogacy performance is the ratio of species to higher taxa (SHR; Bevilacqua et al., 2012; Neeson et al., 2013; van Rijn, Neeson, & Mandelik, 2015). Clearly, this must exhibit some effect, especially when the ratio is low (Gaston, 2000). For the datasets analyzed here, SHR explained almost half of the variation in the correlation between richness of species and higher taxa (“Richness *R²*”). I also observed a significant effect for SHR on *m²* values (the similarity between species‐level and higher taxa ordinations). However, its importance was far less than for richness.

It has also been frequently noted that scale or, more accurately, the degree of species turnover should have an effect on the strength of the surrogacy relationship (Grimbacher et al., 2008; Rosser & Eggleton, 2012). I found no evidence for this affecting the correlation between richness of species and higher taxa. However, it was important for predicting *m²*, in fact more so than SHR, with better surrogacy performance in datasets with high species turnover. Recently, Neeson et al. (2013) used a mathematical model to show that evenness of community structure could also strongly affect the ability of higher taxa to predict patterns of species richness, with better surrogate performance in less even communities. I found no evidence that Richness *R²* depended on *D*_*BP*_. However, it did have a very significant effect on *m²*, with the correspondence between species and higher taxa ordinations declining as the proportional abundance of the dominant species increases. These findings are reassuring with respect to using higher taxa in tropical forest habitats, which are known for their high evenness (Gentry, 1988), and in which the use of surrogates may be particularly beneficial due to their high species richness and poorly known taxonomies.

Neeson et al. (2013) also showed that spurious (i.e., artificial) correlations between richness of species and higher taxa could occur when communities are sampled incompletely, because some samples will by chance contain a greater number of species and so, on average, a greater number of genera and families. Despite this, I found no evidence that Richness *R²* depended on the completeness of the sampling (i.e., the proximity to asymptote of the species accumulation curve). However, the correspondence between species and higher taxa ordinations was higher in communities that had been less intensively sampled (i.e., those further from the asymptote in the species accumulation curve). I suggest that this is because only common species (and the higher taxa to which they belong) are likely to be detected when sampling is incomplete. Ordinations applied to these data will tend to show higher correspondence with higher taxa, because the rarer species within higher taxonomic units do not contribute to the ordination. Finally, I found that when differences in SHR, *D*_*BP*_, DC axis length, and sampling completeness were controlled for, there was no difference in the ability of higher taxa of beetles and ants to predict species richness. However, they did differ very significantly in their ability to predict patterns of community composition; when other predictive variables were controlled for, *m²* values were 0.11 lower on average for beetles than for ants (i.e., the correspondence between species and higher taxa ordinations was higher for beetles than for ants).

It would seem, therefore, that different factors determine the ability of higher taxa to predict species richness and patterns of community composition. For species richness, more than half of the variance in the strength of the surrogacy relationship remained unexplained, whereas for community composition the model explained a remarkable 88%, with taxon (beetles or ants) the most important explanatory variable. I hypothesize that this discrepancy is largely due to differing importance of niche conservation within higher taxa when predicting species richness or assemblage composition. Species richness is measured by simply counting the presence or absence of species in a sample. Thus even when two closely related species with similar ecologies tend to occur in the same samples, stochastic occurrence of single individuals in other samples can lead to low similarity in their distributions. In contrast, the ordination approach applied here accounts for abundance, and the two species would be likely to be seen as responding similarly to the same environmental driver(s). Therefore, the extent to which niches are conserved between related species may strongly influence the ability of higher taxa to predict patterns of community composition, but not richness. The MRPP test supports this hypothesis: I found evidence for species within higher taxa tending to cooccur in beetles, but not in ants. These findings provide an interesting contrast with two previous studies which concluded that niche conservation within higher taxa was not important for surrogacy performance (Bevilacqua et al., 2012; van Rijn et al., 2015).

## 5 Conclusions

Unlike several previous studies, I found that higher taxa of beetles and ants presented unsuitable surrogates for patterns of species composition. The one possible exception was the use of beetle genera, which responded to major environmental gradients in much the same way as species. This poor performance of higher taxa is an important counter to previous examples and illustrates that they should be used with caution. Previous studies on community composition have recommended that SHRs of 2–3 be used as cutoffs for when the surrogacy relationship ceases to be effective (Lovell, Hamer, Slotow, & Herbert, 2007; Melo Carneiro et al., 2010; Timms et al., 2013). However, predicting the strength of the surrogacy relationship using SHR alone seems perilous, and I found both community composition and species turnover to be better predictors. I also found some evidence that the correspondence between ordinations carried out using species and higher taxa was stronger in less well‐sampled communities. Thus while some studies have suggested that higher taxa adequately predict species patterns, their true value as surrogates may be lower than thought. Reassuringly, however, the effect size of sampling completeness was small and was not apparent at all for species richness. On the basis of these results, I therefore make the following recommendations. When applying higher taxa as surrogates for community composition, better performance is expected when SHR is low and in communities with high evenness and high species turnover. However, the most important predictor is the extent to which ecological niches are conserved within higher taxa. In contrast, when predicting patterns of species richness, the importance of stochastic factors in determining surrogacy performance means that reliable predictions can be only reasonably be expected when SHR is very low.

## Conflict of Interest

None declared.

## References

[ece32736-bib-0001] Andersen, A. N. (1995). Measuring more of biodiversity: Genus richness as a surrogate for species richness in Australian ant faunas. Biological Conservation, 73, 39–43.

[ece32736-bib-0002] Balmford, A. , Green, M. J. B. , & Murray, M. G. (1996). Using Higher‐Taxon Richness as a Surrogate for Species Richness: I. Regional Tests. Proceedings of the Royal Society of London B: Biological Sciences, 263, 1267–1274.

[ece32736-bib-0003] Balmford, A. , Jayasuriya, A. H. M. , & Green, M. J. B. (1996). Using Higher‐Taxon Richness as a Surrogate for Species Richness: II. Local Applications. Proceedings of the Royal Society of London B: Biological Sciences, 263, 1571–1575.

[ece32736-bib-0004] Balmford, A. , Lyon, A. J. E. , & Lang, R. M. (2000). Testing the higher‐taxon approach to conservation planning in a megadiverse group: The macrofungi. Biological Conservation, 93, 209–217.

[ece32736-bib-0005] Bartoń, K . (2016). MuMIn: Multi‐Model Inference.

[ece32736-bib-0006] Basset, Y. , Mavoungou, J. F. , Mikissa, J. B. , Missa, O. , Miller, S. E. , Kitching, R. L. , & Alonso, A. (2004). Discriminatory power of different arthropod data sets for the biological monitoring of anthropogenic disturbance in tropical forests. Biodiversity & Conservation, 13, 709–732.

[ece32736-bib-0007] Bates, C. R. , Scott, G. , Tobin, M. , & Thompson, R. (2007). Weighing the costs and benefits of reduced sampling resolution in biomonitoring studies: Perspectives from the temperate rocky intertidal. Biological Conservation, 137, 617–625.

[ece32736-bib-0008] Beccaloni, G. W. , & Gaston, K. J. (1995). Predicting the species richness of neotropical forest butterflies: Ithomiinae (Lepidoptera: Nymphalidae) as indicators. Biological Conservation, 71, 77–86.

[ece32736-bib-0009] Beier, P. , & de Albuquerque, F. S. (2015). Environmental diversity as a surrogate for species representation. Conservation Biology, 29, 1401–1410. 25864466 10.1111/cobi.12495

[ece32736-bib-0010] Berger, W. H. , & Parker, F. L. (1970). Diversity of Planktonic Foraminifera in Deep‐Sea Sediments. Science, 168, 1345–1347. 17731043 10.1126/science.168.3937.1345

[ece32736-bib-0011] Bevilacqua, S. , Terlizzi, A. , Claudet, J. , Fraschetti, S. , & Boero, F. (2012). Taxonomic relatedness does not matter for species surrogacy in the assessment of community responses to environmental drivers. Journal of Applied Ecology, 49, 357–366.

[ece32736-bib-0012] ter Braak, C. J. F. , & Šmilauer, P. (2014). Topics in constrained and unconstrained ordination. Plant Ecology, 216, 683–696.

[ece32736-bib-0013] Brennan, K. E. C. , Ashby, L. , Majer, J. D. , Moir, M. L. , & Koch, J. M. (2006). Simplifying assessment of forest management practices for invertebrates: How effective are higher taxon and habitat surrogates for spiders following prescribed burning? Forest Ecology and Management, 231, 138–154.

[ece32736-bib-0014] Caruso, T. , & Migliorini, M. (2006). Micro‐arthropod communities under human disturbance: Is taxonomic aggregation a valuable tool for detecting multivariate change? Evidence from Mediterranean soil oribatid coenoses. Acta Oecologica, 30, 46–53.

[ece32736-bib-0015] Ellis, D. (1985). Taxonomic sufficiency in pollution assessment. Marine Pollution Bulletin, 16, 459.

[ece32736-bib-0016] Ferla, B. L. , Taplin, J. , Ockwell, D. , & Lovett, J. C. (2002). Continental scale patterns of biodiversity: Can higher taxa accurately predict African plant distributions? Botanical Journal of the Linnean Society, 138, 225–235.

[ece32736-bib-0017] Gaston, K. J. (2000). Biodiversity: Higher taxon richness. Progress in Physical Geography, 24, 117–127.

[ece32736-bib-0018] Gaston, K. J. , & Blackburn, T. M. (1995). Mapping Biodiversity Using Surrogates for Species Richness: Macro‐Scales and New World Birds. Proceedings of the Royal Society of London B: Biological Sciences, 262, 335–341.

[ece32736-bib-0019] Gaston, K. J. , & Williams, P. H. (1993). Mapping the World's Species‐The Higher Taxon Approach. Biodiversity Letters, 1, 2–8.

[ece32736-bib-0020] Gentry, A. H. (1988). Tree species richness of upper Amazonian forests. Proceedings of the National Academy of Sciences, 85, 156–159. 10.1073/pnas.85.1.156 PMC279502 16578826

[ece32736-bib-0021] Gower, J. C. (1971). Statistical methods of comparing different analyses of the same data In Hodson F. R., Kendall D. G., & Tautu P. (Eds.), Mathematics in the archeological and historical sciences (pp. 138–149). Edinburgh: University Press.

[ece32736-bib-0022] Grimbacher, P. S. , Catterall, C. P. , & Kitching, R. L. (2008). Detecting the effects of environmental change above the species level with beetles in a fragmented tropical rainforest landscape. Ecological Entomology, 33, 66–79.

[ece32736-bib-0023] Heino, J. , & Soininen, J. (2007). Are higher taxa adequate surrogates for species‐level assemblage patterns and species richness in stream organisms? Biological Conservation, 137, 78–89.

[ece32736-bib-0024] Hill, M. O. , & Gauch, H. G. (1980). Detrended correspondence analysis: An improved ordination technique. Vegetatio, 42, 47–58.

[ece32736-bib-0025] Jackson, D. A. , & Somers, K. M. (1991). Putting Things in Order: The Ups and Downs of Detrended Correspondence Analysis. The American Naturalist, 137, 704–712.

[ece32736-bib-0026] Jew, E. K. K. , Loos, J. , Dougill, A. J. , Sallu, S. M. , & Benton, T. G. (2015). Butterfly communities in miombo woodland: Biodiversity declines with increasing woodland utilisation. Biological Conservation, 192, 436–444.

[ece32736-bib-0027] Jones, F. C. (2008). Taxonomic sufficiency: The influence of taxonomic resolution on freshwater bioassessments using benthic macroinvertebrates. Environmental Reviews, 16, 45–69.

[ece32736-bib-0028] Kallimanis, A. S. , Mazaris, A. D. , Tsakanikas, D. , Dimopoulos, P. , Pantis, J. D. , & Sgardelis, S. P. (2012). Efficient biodiversity monitoring: Which taxonomic level to study? Ecological Indicators, 15, 100–104.

[ece32736-bib-0029] Lovell, S. , Hamer, M. , Slotow, R. , & Herbert, D. (2007). Assessment of congruency across invertebrate taxa and taxonomic levels to identify potential surrogates. Biological Conservation, 139, 113–125.

[ece32736-bib-0030] Melo Carneiro, F. , Bini, L. M. , & Rodrigues, L. C. (2010). Influence of taxonomic and numerical resolution on the analysis of temporal changes in phytoplankton communities. Ecological Indicators, 10, 249–255.

[ece32736-bib-0031] Nahmani, J. , Lavelle, P. , & Rossi, J.‐P. (2006). Does changing the taxonomical resolution alter the value of soil macroinvertebrates as bioindicators of metal pollution? Soil Biology and Biochemistry, 38, 385–396.

[ece32736-bib-0032] Nakagawa, S. , & Schielzeth, H. (2013). A general and simple method for obtaining *R^2^* from generalized linear mixed‐effects models. Methods in Ecology and Evolution, 4, 133–142.

[ece32736-bib-0033] Nakamura, A. , Catterall, C. P. , House, A. P. N. , Kitching, R. L. , & Burwell, C. J. (2006). The use of ants and other soil and litter arthropods as bio‐indicators of the impacts of rainforest clearing and subsequent land use. Journal of Insect Conservation, 11, 177–186.

[ece32736-bib-0034] Neeson, T. M. , Van Rijn, I. , & Mandelik, Y. (2013). How taxonomic diversity, community structure, and sample size determine the reliability of higher taxon surrogates. Ecological Applications, 23, 1216–1225. 23967587 10.1890/12-1167.1

[ece32736-bib-0035] Oksanen, J. , Blanchet, F. G. , Kindt, R. , Legendre, P. , Minchin, P. R. , O'Hara, R. B. , … Wagner, H. (2013). vegan: Community Ecology Package. R package version 2.0‐6. https://CRAN.R-project.org/package=vegan.

[ece32736-bib-0036] Oliver, I. , & Beattie, A. J. (1996). Invertebrate Morphospecies as Surrogates for Species: A Case Study. Conservation Biology, 10, 99–109.

[ece32736-bib-0037] Pik, A. J. , Dangerfield, J. M. , Bramble, R. A. , Angus, C. , & Nipperess, D. A. (2002). The Use of Invertebrates to Detect Small‐scale Habitat Heterogeneity and its Application to Restoration Practices. Environmental Monitoring and Assessment, 75, 179–199. 12002286 10.1023/a:1014444032375

[ece32736-bib-0038] Pik, A. J. , Oliver, I. , & Beattie, A. J. (1999). Taxonomic sufficiency in ecological studies of terrestrial invertebrates. Australian Journal of Ecology, 24, 555–562.

[ece32736-bib-0039] Pinheiro, J. , Bates, D. , DebRoy, S. , & Sarkar, D ., & the R Development Core Team . (2016). nlme: Linear and Nonlinear Mixed Effects Models.

[ece32736-bib-0040] Prance, G. T. (1994). A Comparison of the Efficacy of Higher Taxa and Species Numbers in the Assessment of Biodiversity in the Neotropics. Philosophical Transactions of the Royal Society of London B: Biological Sciences, 345, 89–99.

[ece32736-bib-0041] Prinzing, A. , Klotz, S. , Stadler, J. , & Brandl, R. (2003). Woody plants in Kenya: Expanding the Higher‐Taxon Approach. Biological Conservation, 110, 307–314.

[ece32736-bib-0042] R Core Team . (2013). R: A language and environment for statistical computing. Vienna, Austria; R Foundation for Statistical Computing.

[ece32736-bib-0043] van Rijn, I. , Neeson, T. M. , & Mandelik, Y. (2015). Reliability and refinement of the higher taxa approach for bee richness and composition assessments. Ecological Applications, 25, 88–98. 26255359 10.1890/13-2380.1

[ece32736-bib-0044] Rodrigues, A. S. L. , & Brooks, T. M. (2007). Shortcuts for Biodiversity Conservation Planning: The Effectiveness of Surrogates. Annual Review of Ecology, Evolution, and Systematics, 38, 713–737.

[ece32736-bib-0045] Rosser, N. , & Eggleton, P. (2012). Can higher taxa be used as a surrogate for species‐level data in biodiversity surveys of litter/soil insects? Journal of Insect Conservation, 16, 87–92.

[ece32736-bib-0046] Schnell, M. R. , Pik, A. J. , & Dangerfield, J. M. (2003). Ant community succession within eucalypt plantations on used pasture and implications for taxonomic sufficiency in biomonitoring. Austral Ecology, 28, 553–565.

[ece32736-bib-0047] Schönemann, P. H. , & Carroll, R. M. (1971). Fitting one matrix to another under choice of a central dilation and a rigid motion. Psychometrika, 35, 245–255.

[ece32736-bib-0048] Timms, L. L. , Bowden, J. J. , Summerville, K. S. , & Buddle, C. M. (2013). Does species‐level resolution matter? Taxonomic sufficiency in terrestrial arthropod biodiversity studies. Insect Conservation and Diversity, 6, 453–462.

[ece32736-bib-0049] Warton, D. I. , & Hui, F. K. C. (2011). The arcsine is asinine: The analysis of proportions in ecology. Ecology, 92, 3–10. 21560670 10.1890/10-0340.1

[ece32736-bib-0050] Warwick, R. M. (1988). The level of taxonomic discrimination required to detect pollution effects on marine benthic communities. Marine Pollution Bulletin, 19, 259–268.

[ece32736-bib-0051] Warwick, R. M. (1993). Environmental impact studies on marine communities: Pragmatical considerations. Australian Journal of Ecology, 18, 63–80.

[ece32736-bib-0052] Wright, I. A. , Chessman, B. C. , Fairweather, P. G. , & Benson, L. J. (1995). Measuring the impact of sewage effluent on the macroinvertebrate community of an upland stream: The effect of different levels of taxonomic resolution and quantification. Australian Journal of Ecology, 20, 142–149.

